# Effects of Astaxanthin, Lutein, and Zeaxanthin on Eye–Hand Coordination and Smooth-Pursuit Eye Movement after Visual Display Terminal Operation in Healthy Subjects: A Randomized, Double-Blind Placebo-Controlled Intergroup Trial

**DOI:** 10.3390/nu15061459

**Published:** 2023-03-17

**Authors:** Keisuke Yoshida, Osamu Sakai, Tomoo Honda, Tomio Kikuya, Ryuji Takeda, Akiyoshi Sawabe, Masamaru Inaba, Chieko Koike

**Affiliations:** 1 Senju Pharmaceutical, Co., Ltd., Osaka 541-0048, Japan; 2 Department of Nutritional Sciences for Well-Being, Faculty of Health Sciences for Welfare, Kansai University of Welfare Sciences, Osaka 582-0026, Japan; 3 Department of Applied Biological Chemistry, Faculty of Agriculture, Kindai University, Nara 631-8505, Japan; 4 Inaba Eye Clinic, Osaka 530-0001, Japan; 5 College of Pharmaceutical Sciences, Ritsumeikan University, Shiga 525-8577, Japan; 6 Center for Systems Vision Science, Research Organization of Science and Technology, Ritsumeikan University, Shiga 525-8577, Japan

**Keywords:** visual display terminals (VDT), astaxanthin, lutein, zeaxanthin, eye–hand coordination, smooth-pursuit eye movements, macular pigment optical density (MPOD)

## Abstract

(1) Background: The impairment of eye–hand coordination and smooth-pursuit eye movement caused by visual display terminal (VDT) operation is thought to impair daily living activities, for which no effective methods are currently known. On the other hand, various food ingredients, including astaxanthin, lutein, and zeaxanthin, are known to help improve the eye health of VDT operators. This study aimed to test the hypothesis that the combination of astaxanthin, lutein, and zeaxanthin can prevent the impairment of eye–hand coordination and smooth-pursuit eye movement caused by VDT operation. (2) Methods: We conducted a randomized, placebo-controlled, parallel-group clinical trial. Healthy subjects who regularly worked with VDTs were randomly assigned to the active and placebo groups. All of the subjects took soft capsules containing 6 mg of astaxanthin, 10 mg of lutein, and 2 mg of zeaxanthin or placebo soft capsules once daily for eight weeks. We evaluated the eye–hand coordination, smooth-pursuit eye movements, and macular pigment optical density (MPOD) at 0, two, four, and eight weeks after soft-capsule intake. (3) Results: The active group showed significantly improved eye–hand coordination after VDT operation at eight weeks. However, there was no clear improvement in the effect of the supplementation on smooth-pursuit eye movements. The active group also showed a significant increase in MPOD levels. (4) Conclusions: Consumption of a supplement containing astaxanthin, lutein, and zeaxanthin mitigates the decline of eye–hand coordination after VDT operation.

## 1. Introduction

In recent years, the use of visual display terminals (VDTs), including computers, smartphones, tablet devices, and video game consoles, has become a widespread and essential part of the modern lifestyle. In particular, during the coronavirus disease 2019 pandemic and the period of social isolation, people were forced to work from home and communicate using these devices. VDTs have made our lives more convenient and efficient; however, VDT operation leads to a decline in various visual functions. Prolonged VDT operations cause transient myopia due to the requirement for sustained effort for accommodation [1]. Furthermore, previous studies have shown that VDT operation induces changes in oculomotor function through extraocular muscle strain [2], and playing video games using VDTs involving horizontal eye movement temporarily reduces dynamic visual activity [3]. VDT operation thus has a negative impact on visual function, which is essential in daily activity.

Eye–hand coordination is the ability of our vision system to coordinate the information received through the eyes to control, guide, and direct the hands [4]. In other words, eye–hand coordination ability is required to gaze at target objects quickly and accurately and to reach the hand to the target. In everyday life, intervention for eye–hand coordination becomes necessary to successfully perform activities of daily living, such as reaching for a cup on the table, tapping a smartphone to surf the internet, typing letters on a keyboard, driving a car, and playing tennis. Eye–hand coordination typically requires rapid eye movements called saccades. Saccades are discrete movements that rapidly change the orientation of the eye, thereby translating an image of the object of interest from an eccentric retinal location to the fovea. Smooth-pursuit eye movements occur in other visual functions. Smooth-pursuit eye movement supports vision by holding the image of a moving target on the fovea [5] and is an integral part of everyday activities, such as tracking the mouse cursor while working on a computer, tracking an approaching car, and tracking a tennis ball. In daily life, we use a combination of saccade and pursuit eye movements to stabilize the retinal images of selected objects within the fovea.

Conversely, disruption to eye-–hand coordination and smooth-pursuit eye movement due to aging [6,7], disease [8,9,10], injury [11], and developmental disorders [12] leads to considerable degeneration in the productivity and quality of life (QOL). VDT operation is considered to disrupt eye–hand coordination and smooth-pursuit eye movement because the VDT operation is attributed to a decline in accommodation and oculomotor function, which are related to eye–hand coordination and smooth-pursuit eye movement. In April 2013, Japan’s Ministry of Health, Labour and Welfare promoted “The second term of National Health Promotion Movement in the Twenty-First Century (Health Japan 21 (the second term))”, which describes basic goals for the implementation of national health promotion in detail. Maintaining and improving the functions necessary for engaging in social life is the goal. In modern society, where VDT operations have become increasingly common, the fact that VDT operations impair eye–hand coordination and smooth-pursuit eye movement might become a serious problem. Therefore, solving this problem may help achieve the goal of protecting national health. However, no previous studies have verified improvement methods in eye–hand coordination and smooth-pursuit eye movement impaired by VDT operation.

Some studies have shown that various food ingredients, such as astaxanthin, lutein, and zeaxanthin, prevent visual function decline induced by VDT [13,14,15]. Astaxanthin is a naturally occurring red carotenoid pigment that belongs to the family of secondary xanthophylls [16]. It is typically found in marine environments, especially in microalgae and seafood, such as salmonids, shrimp, and crabs. Astaxanthin possesses an antioxidant effect 100–1000 times stronger than that of vitamin E and 40 times stronger than that of β-carotene [17,18]. Regarding its effect on the eye, astaxanthin is reported to reduce the degradation of nitric oxide, which is involved in the dilatation of blood vessels [19] and significantly increases the retinal capillary blood flow near the optic disc [20]. This report suggests that astaxanthin improves blood circulation and ciliary body relaxation. Furthermore, astaxanthin inhibits the nuclear factor-kappa B signaling pathway in the ciliary body [21]. According to previous clinical studies, astaxanthin consumption restores the accommodative function and improves ocular symptoms in VDT operators [13,14].

Lutein and zeaxanthin are fat-soluble antioxidants belonging to the xanthophyll class. They are abundant in eggs and dark green leafy vegetables, such as kale and spinach [22,23,24]. In the human body, lutein and zeaxanthin are mainly distributed in the macula, which is the center of the retina. They compose the macular pigment, and macular pigment optical density (MPOD) has been widely used as an indicator to quantify lutein/zeaxanthin in the macular [25,26]. Thus, they are thought to protect the retina from damage by absorbing blue light. A previous study showed that lutein administration to rhesus monkeys increased MPOD and reduced eye damage caused by blue light [27]. Furthermore, lutein intake by VDT operators increases MPOD levels and improves chromatic contrast and recovery from photostress [28].

As mentioned above, astaxanthin, lutein, and zeaxanthin are known to have many health benefits. Therefore, we hypothesized that dietary supplementation with astaxanthin, lutein, and zeaxanthin has substantial potential for preventing eye–hand coordination and smooth-pursuit eye movements impairment induced by VDT. In this randomized, double-blind, parallel-group study, we investigated the effects of astaxanthin, lutein, and zeaxanthin on visual function following VDT activity. 

## 2. Materials and Methods

### 2.1. Study Design

This was a randomized, double-blind, placebo-controlled study. The study protocol was approved by the independent ethics committee of the Kobuna Orthopedic Clinic on 28 February 2022 (approval no. MK-2202-01) and registered in the University Hospital Medical Information Network Clinical Trials Registry (UMIN-CTR; UMIN000047137). The study was performed in compliance with ethical principles based on the Declaration of Helsinki and the Ethical Guidelines for Epidemiological Studies (Notification of the Ministry of Education, Culture, Sports, Science and Technology and the Ministry of Health, Labour and Welfare). The clinical trial was conducted from 28 March to 2 July 2022 at the Japan Sports Vision Association (Tokyo, Japan).

### 2.2. Subjects

Healthy Japanese males and females aged 20–60 years who met the inclusion criteria participated in the study. The inclusion criteria were as follows: (1) subjects with a distant bilateral vision of 0.6 or higher (uncorrected or corrected); (2) subjects who routinely played video games, used computers, or performed VDT activities; (3) subjects who could undergo examinations and observations as scheduled; and (4) subjects who received sufficient explanation of the purpose and content of the trial, had the ability to consent, volunteered to participate after understanding the trial fully, and agreed to participate in the trial in writing. The exclusion criteria were as follows: (1) medical history of serious diseases, such as liver, renal, cardiovascular, gastrointestinal, respiratory, hematological, autoimmune, endocrine system, and metabolic disease; (2) history of drug allergy or serious food allergy; (3) continuous use of medicines, health foods, or supplements that may affect the study; and (4) subjects judged by the investigator as inappropriate for the study.

### 2.3. Sample Size

Before conducting this study, the degree of decrease in eye–hand coordination between pre- and post-VDT operations was preliminarily examined. Consequently, a decrease of approximately 3% was observed. Based on this, we determined that the ingestion of our test food would result in a reduction of approximately 1% compared with the placebo, with a standard deviation of 1.5%. The sample size was calculated assuming 80% power and was evaluated in 29 subjects per group. Furthermore, considering the possibility of dropouts, the number of participants per group was planned to be 32.

### 2.4. Intervention

The test soft capsules included 6 mg astaxanthin, 10 mg lutein, and 2 mg zeaxanthin (66 mg/capsule: Hematococcus-pluvialis-derived pigment [BGG Japan Co., Ltd., Tokyo, Japan) and 55 mg/capsule: marigold [BGG Japan Co., Ltd.]). The placebo was a soft capsule containing rice oil instead of these ingredients. The subjects took either one test soft capsule (active group) or one placebo capsule (placebo group) daily for eight weeks. According to previous clinical studies, 6 mg/day astaxanthin consumption restores the accommodative function and improves ocular symptoms in VDT operators [14]. Furthermore, 10 mg/day lutein and 2 mg/day zeaxanthin consumption increases the contrast sensitivity in healthy subjects with VDT use [28]. The doses of astaxanthin, lutein, and zeaxanthin in this study were decided regarding reports from these clinical trials.

### 2.5. Schedule

The study schedule is presented in Table 1. The subjects were recruited from volunteers (i.e., those who were registered with the Global Sense Co., Ltd., Tokyo, Japan), based on the inclusion and exclusion criteria. Before starting the screening process, the participants were fully informed of the contents and methods of the study and their written consent was obtained. The subjects visited the site (the Japan Sports Vision Association) and underwent an examination before ingestion (screening test, 0 weeks) and after two, four, and eight weeks of intake. Visual function (eye–hand coordination and smooth-pursuit eye movements) and QOL were evaluated before and after the VDT operation. An MPOD test was performed before the VDT operation.

### 2.6. VDT Operation

The VDT operation consisted of playing a video game with a smartphone for 30 min. On the smartphone screen, 41 identical kanji characters and one similar but different kanji character were displayed (seven vertical by six horizontal; 42 characters in total). The participants tapped on the different ones displayed as soon as they found them. If the subject tapped correctly, another task (another 42 kanji characters) was displayed, and the subject repeated the task (finding and tapping different kanji characters). Additionally, a 30 cm cord was attached to the smartphone, and the subjects wore the cord around their necks to maintain a constant distance between their eyes and the smartphone while performing the VDT operation.

### 2.7. Randomization

This was a double-blind study. An individual not involved in the evaluation of the study was designated as the allocation manager. The allocation manager checked the indistinguishability of each test item, allocated it, and was blinded to the test items. The allocation manager prepared an allocation table using the substitution block method to ensure that there was no bias between age, sex, and change in the eye–hand coordination time pre- and post-VDT operation during the screening test in the two groups. The allocation tables were strictly sealed and not opened until the participants were analyzed.

### 2.8. Primary Outcome

The primary outcomes were visual functions (eye–hand coordination time and accuracy rate and visual motion reaction time through smooth-pursuit eye movement).

Eye–hand coordination was measured using V-training 2G (Tokyo Optical Co., Ltd., Saitama, Japan). The V-training 2G was equipped with a monitor (inner dimensions: length 610 mm and width 1080 mm), which was adjusted to achieve the appropriate height and distance (30 cm) for each subject. At the start of the measurement, a target (a square of 22 mm in height and 22 mm in width) was displayed at random positions on the monitor, and the subjects quickly found the target with both eyes and accurately pointed to its center with their fingertips. After they had accurately pointed, the next target was randomly displayed on the monitor, and the subject again pointed to the target quickly. This task was repeated until 30 targets were identified. As an evaluation method, the time taken to finish pointing at the 30 targets was measured and designated as the eye–hand coordination time. The percentage of the 30 targets that were touched correctly was evaluated as the eye–hand coordination accuracy rate. The eye–hand coordination time and accuracy rate were evaluated three times, before and after the VDT operation, and the average value was calculated and used for evaluation.

Smooth-pursuit eye movements were measured using V-training 2G. At the beginning of the measurement, a black circular target moved quickly (approximately 80 mm/s) and randomly on a V-training 2G monitor. The subject tracked the moving target with both eyes without moving their head while keeping their finger on the measurement button on the monitor. During this process, the color of the target changed from black to white at random times, and the subjects removed their fingers from the measurement button as soon as the color changed. The target color was changed three times during each set of evaluations. The time spent from the target color change to the release of the measurement button was evaluated as the visual motion reaction time for smooth-pursuit eye movements. This evaluation was conducted three times, before and after the VDT operation, and we calculated the average change in visual motion reaction time between the pre- and post-VDT operations.

### 2.9. Secondary Outcomes 

#### 2.9.1. MPOD

The MPOD levels were measured using a macular pigment screen (MPS2; M.E. Technica Co., Ltd., Kyoto, Japan). MPS2 is a heterochromatic flicker photometry instrument [29]. The blue and green light wavelengths were 465 and 530 nm, respectively. The size of the light plane was 1° in diameter; thus, the MPOD obtained with this instrument is 0.5° from the foveal center [30]. The subjects were asked to watch a light plane and push a button on the machine immediately after observing flickering. MPOD levels in the left and right eyes were measured, and the change in the average MPOD levels in both eyes from week 0 was calculated. 

#### 2.9.2. QOL Questionnaire

The subjects completed QOL questionnaires using a visual analog scale (VAS) to assess subjective symptoms. In the VAS method, a 100 mm-long horizontal line was printed, where the left end (0 mm) was defined as the worst condition and the right end (100 mm) was defined as the best condition. The participants expressed their feelings about their QOL. The VAS assessment was conducted pre- and post-VDT operation.

### 2.10. Safety Evaluation

The following safety endpoints were established. Body weight, body mass index (BMI), and blood pressure were measured during physical examinations. Fluctuations in body weight, blood pressure, and BMI were observed during the intake period to check for any abnormal changes; the subjects recorded a web diary during the weeks of the intake period, and all adverse events that occurred were analyzed and assessed for association with the test food.

### 2.11. Statistical Analysis

SAS9.4 (SAS Inc., Cary, NC, USA) was used for statistical analysis. Primary endpoint comparisons were analyzed using the Student’s *t*-test, and for secondary outcomes, intergroup comparisons were analyzed using the Student’s *t*-test. The tests were applied assuming the normality of each dataset (multiplicity was not considered) with a significance level of 5% (two-tailed test).

## 3. Results

### 3.1. Subject Background

**Figure 1:**
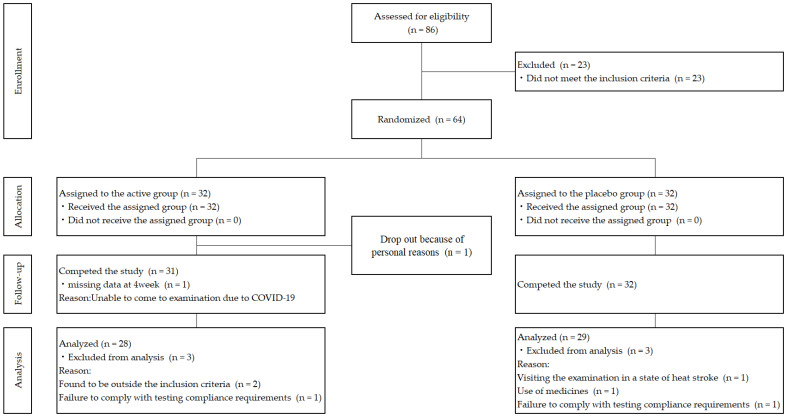
Flow diagram of the participants.

A flow diagram of the participants in this study is shown in Figure 1. A total of 86 subjects underwent screening and pre-trial tests, of whom 64 were chosen to participate in the study. Each of the 64 participants were then randomly assigned to either the active or placebo group by the allocation manager. During the trial, one active group participant dropped out for personal reasons and the remaining 63 participants completed the study. After we had reviewed the subjects, three subjects in the active group were excluded from the analysis for reasons outside the inclusion criteria (*n* = 2) and failure to comply with the testing requirements (*n* = 1). Furthermore, three subjects in the placebo group were excluded from the analysis because they could not visit the examination site due to heat stroke (*n* = 1) or use of medicines (*n* = 1). Thus, 28 and 29 subjects in the active and placebo groups, respectively, were included in the analysis. The backgrounds of the study participants are listed in Table 2. There were no significant differences in the background factors between the two groups.

### 3.2. Eye–Hand Coordination

The results of the eye–hand coordination tests are shown in Table 3. For the post-VDT operation, eye–hand coordination times at eight weeks in the active group were significantly shorter than those of the placebo group (active group 21.45 ± 1.59 s, placebo group 22.53 ± 1.76 s; *p* = 0.019). Furthermore, the eye–hand coordination accuracy rate for post-VDT operation at eight weeks in the active group was significantly higher than that of the placebo group (active group 83.72 ± 6.51%, placebo group 77.30 ± 8.55%; *p* = 0.002). In addition, the change in the eye–hand coordination time between the pre- and post-VDT operations at eight weeks in the active group was significantly lower than that in the placebo group (active group 0.05 ± 1.39 s, placebo group 0.81 ± 1.37 s; *p* = 0.041). The change in the eye–hand coordination accuracy rate between the pre- and post VDT operations at eight weeks in the active group was significantly higher than that of the placebo group (active group 0.77 ± 6.19%, placebo group −3.14 ± 6.58%; *p* = 0.025). These results showed that the improvement effect of the test food on eye–hand coordination function was decreased by the VDT operation.

### 3.3. Smooth-Pursuit Eye Movements

The results of the visual motion reaction time through smooth-pursuit eye movements are shown in Table 4. The change in the reaction time pre- and post-VDT operation at two weeks in the active group was significantly lower than that in the placebo group (active group, 0.016 ± 0.156; placebo group, 0.104 ± 0.149; *p* = 0.033). However, the changes in smooth-pursuit eye movements between pre- and post-VDT operations at four and eight weeks in the active group were not significantly different from those in the placebo group.

### 3.4. The Results of MPOD Levels

The MPOD values are listed in Table 5. The change in MPOD levels between the baseline and eight weeks in the active group was significantly higher than those in the placebo group (active group, 0.015 ± 0.052; placebo group, −0.016 ± 0.052; *p* = 0.029).

### 3.5. QOL Questionnaire

The QOL questionnaire revealed no significant differences between the groups.

### 3.6. Safety Assessment

No medically problematic height, body weight, BMI, and blood pressure changes were observed during the study period. Analysis of the web diary recorded by the subjects revealed no adverse events causally related to intake of the test food.

## 4. Discussion

The major finding of the present study was that VDT operation temporarily impaired the eye–hand coordination and smooth-pursuit eye movements, and astaxanthin, lutein, and zeaxanthin improved eye–hand coordination impaired by VDT operation. This finding supports the hypothesis that dietary supplementation with astaxanthin, lutein, and zeaxanthin is of benefit in preventing eye–hand coordination impairment caused by VDT. VDT operation has been reported to lead to continuous tension of the ciliary muscle because the time spent looking closely at a terminal is long, leading to a decrease in the amplitude of accommodation [31]. In addition, VDT operation induces a decline in oculomotor function through extraocular muscle strain [5]. Therefore, we theorize that temporary eye–hand coordination and smooth-pursuit eye movement impaired by VDT operation may be attributed to a decline in accommodation and oculomotor function. Moreover, VDT operation not only decreases visual function but also causes musculoskeletal symptoms, such as stiffness of the shoulders and back [3]. According to a previous study, prolonged seated immobility during VDT operation reduces blood velocity and delays reaction time during working memory performance [32]. Therefore, we theorize that physical functions impaired by VDT operation may decrease eye–hand coordination function because eye–hand coordination frequently involves physical actions.

The following were considered concerning the action mechanism of the test food on eye–hand coordination. Astaxanthin consumption has been reported to significantly restore accommodative function [12,13] through blood improvement, muscular damage control, and ciliary body smooth muscle relaxation [23]. Furthermore, we assume that astaxanthin may also restore extraocular muscle through these effects, improving oculomotor function, such as saccades. Moreover, astaxanthin consumption has been shown to significantly improve musculoskeletal symptoms, such as shoulder and back stiffness, in VDT operators [23]. Therefore, we believe that astaxanthin may improve eye–hand coordination after VDT operation by improving accommodative, oculomotor, and physical functions. 

In the present study, the change in MPOD levels between baseline and eight weeks in the active treatment group was significantly higher than that in the placebo group. The macular pigment is composed of lutein and zeaxanthin, and MPOD is used to qualify lutein and zeaxanthin in the macular [25,26]. Therefore, these results indicates that lutein and zeaxanthin accumulate in the retinal macula after intake of the test food. Furthermore, this concurs with a previous study that reported that eight weeks of lutein-rich spinach intake increased MPOD levels [33]. Lutein and zeaxanthin absorb the blue light that VDT exposes to the retinal macula. According to a previous study, the consumption of lutein and zeaxanthin increases contrast sensitivity in healthy subjects with VDT use [28]. Additionally, lutein and zeaxanthin have been reported to be present not only in the retina but also in the visual and motor cortices, which influence visual–motor responses [34]. A previous study indicated that MPOD is positively correlated with the visual motor response and temporal contrast sensitivity function, which are involved in visual processing speed [34,35]. Moreover, intervention with lutein and zeaxanthin is expected to increase processing speed, even in young healthy subjects [36]. The detailed mechanism of action is unclear, although it is thought that lutein and zeaxanthin act to create structural changes within or across neurons or glia by enhancing gap junction communication, which leads to a lasting improvement in processing speed [37]. Although there was no observation of the enhancing effect of the test coordination pre-VDT operation in the present study, we speculate that lutein and zeaxanthin can maintain eye–hand coordination post-VDT by increasing visual processing speed and contrast sensitivity. 

In the present study, although consumption of the test food improved smooth-pursuit eye movements impaired by VDT operation at two weeks, there was no observation of the improvement effects of the test food on smooth-pursuit eye movements at eight weeks. This may be because the VDT operation method in this study mainly used saccade eye movements and the range of decrease in smooth-pursuit eye movements induced by VDT operation was too small for accurate evaluation. In addition, saccade and smooth-pursuit eye movements traditionally have distinct neural systems. For smooth-pursuit eye movement, these pathways are composed of a seemingly simple circuit connecting areas in the temporal and frontal lobes of the cerebral cortex with pursuit-related motor regions of the cerebellum [37,38,39]. However, the saccadic pathway has several additional routes that are not imparted to the pursuit system [40,41]. Although details of the cause are not clear, the different time points of the effect observed on eye–hand coordination and smooth-pursuit eye movements may be due to these distinct neural systems. Further studies are necessary to clarify the effects of the test food on smooth-pursuit eye movements.

Regarding the QOL questionnaire, for one of the secondary outcomes, there was no observation of improvement effects of the test food. The VAS method is subject to high variability, because individuals often feel differently about questions. Thus, as the number of participants in this study was small, it may not have been accurately evaluated.

The test food can contribute to enhancing QOL by suppressing the decline in eye–hand coordination after VDT. Moreover, this study has suggested that test foods can contribute to PC game and sports performance. Previous studies have proposed that strenuous exercise impairs peripheral visual perception by decreasing cerebral oxygenation [42,43]. We theorize that strenuous exercise may impair the eye–hand coordination function and that the test food can maintain the eye–hand coordination of various athletes, such as tennis players, baseball players, and electronic sports players. 

Our study had several limitations. First, astaxanthin, lutein, and zeaxanthin are present in daily meals. In this study, we prohibited the participants from consuming health foods or supplements that contained astaxanthin, lutein, and zeaxanthin. However, we did not impose strict dietary restrictions on our subjects, and it is unclear what amount of these ingredients they consumed in their daily diet. Thus, the effects of supplemental astaxanthin, lutein, and zeaxanthin may have been obscured. Second, in this study, the combination of astaxanthin, lutein, and zeaxanthin was designed as the active group rather than astaxanthin or lutein/zeaxanthin alone. Thus, it is unclear whether the effect of the test food on eye–hand coordination is due to additive or synergistic effects. Although we believe that the combination of these nutrients is essential for the effects on eye–hand coordination because of the different mechanisms of action between astaxanthin and lutein/zeaxanthin, further studies are necessary to clarify the mechanisms underlying the beneficial effects of these nutrients.

## 5. Conclusions

The effect of the intake of test foods containing astaxanthin, lutein, and zeaxanthin on eye–hand coordination during VDT operation in healthy Japanese adult subjects was investigated in this study. Consumption of the test foods improved eye–hand coordination function impaired by VDT operation. Furthermore, consumption of the test foods was found to be safe under the conditions of this study.

## Figures and Tables

**Table 1:** Schedule of enrollment, intervention, and assessment.

Item		Enrollment	Test Period			
			0 Weeks	2 Weeks	4 Weeks	8 Weeks
Informed consent		●				
Background investigation		●				
Selection			●			
Allocation			●			
Physical examinations			●	●	●	●
Visual function test	Eye–hand coordination		●●	●●	●●	●●
	Smooth pursuit eye movement		●●	●●	●●	●●
QOL questionnaire			●●	●●	●●	●●
MPOD			●	●	●	●
Ingestion of test foods or placebo				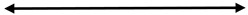		
Diary record				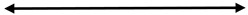		

● Implementation, ●● Implementation twice (pre- and post-VDT operation); 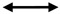 Daily practice.

**Table 2:** Baseline characteristics of the participants who completed the eight-week study.

Item		Active Group (*n* = 28)	Placebo Group (*n* = 29)
Age (years)		31.1 ± 8.2	30.1 ± 8.6
Sex (Male/Female)		25/3	27/2
BMI (kg/m^2^)		23.0 ± 4.0	22.2 ± 4.1
change of visual function pre- and post-VDT operation	Eye−hand coordination time (s)	1.10 ± 0.93	1.32 ± 2.16
	Eye−hand coordination accuracy rate (%)	−4.77 ± 6.03	−6.34 ± 12.0
	Smooth pursuit eye movement (s)	0.066 ± 0.115	0.120 ± 0.127
MPOD		0.576 ± 0.151	0.581 ± 0.136

Data are presented as the mean ± SD.

**Table 3:** The results of the eye–hand coordination tests.

	Baseline			2 Weeks			4 Weeks			8 Weeks		
	Active Group (*n* = 28)	Placebo Group (*n* = 29)	*p* Value *	Active Group (*n* = 28)	Placebo Group (*n* = 29)	*p* Value *	Active Group (*n* = 27)	Placebo Group (*n* = 29)	*p* Value *	Active Group (*n* = 28)	Placebo Group (*n* = 29)	*p* Value *
Time												
Pre-VDT operation (s)	22.53 ± 1.57	22.92 ± 1.40	0.318	22.42 ± 1.27	22.78 ± 1.95	0.406	21.67 ± 1.55	22.09 ± 1.94	0.382	21.40 ± 1.23	21.72 ± 1.69	0.424
Post-VDT operation (s)	23.62 ± 1.63	24.24 ± 2.41	0.262	22.64 ± 1.44	23.37 ± 1.47	0.064	22.12 ± 1.66	22.43 ± 1.53	0.473	21.45 ± 1.59	22.53 ± 1.76	0.019
Change between pre- and post-VDT operation (s)	1.10 ± 0.93	1.32 ± 2.16	0.613	0.23 ± 1.06	0.59 ± 1.60	0.320	0.45 ± 1.28	0.34 ± 1.30	0.756	0.05 ± 1.39	0.81 ± 1.37	0.041
Accuracy rate												
Pre-VDT operation (%)	80.71 ± 6.29	78.17 ± 7.41	0.170	81.59 ± 5.30	77.71 ± 9.38	0.061	82.13 ± 4.93	80.35 ± 9.56	0.390	82.95 ± 5.31	80.44 ± 7.03	0.135
Post-VDT operation (%)	75.94 ± 7.84	71.83 ± 12.7	0.149	79.45 ± 5.55	75.26 ± 8.61	0.034	80.92 ± 6.72	78.80 ± 8.71	0.314	83.72 ± 6.51	77.30 ± 8.55	0.002
Change between pre- and post- VDT operation (%)	−4.77 ± 6.03	−6.34 ± 12.0	0.536	−2.15 ± 6.59	−2.46 ± 8.32	0.877	−1.21 ± 6.04	−1.55 ± 6.24	0.836	0.77 ± 6.19	−3.14 ± 6.58	0.025

Data are presented as the mean ± SD. * Student’s *t*-test, vs the placebo group.

**Table 4:** The results of smooth-pursuit eye movement tests.

	Baseline			2 Weeks			4 Weeks			8 Weeks		
	Active Group (*n* = 28)	Placebo Group (*n* = 29)	*p* Value *	Active Group (*n* = 28)	Placebo Group (*n* = 29)	*p* Value *	Active Group (*n* = 27)	Placebo Group (*n* = 29)	*p* Value *	Active Group (*n* = 28)	Placebo Group (*n* = 29)	*p* Value *
Pre-VDT operation (s)	1.368 ± 0.207	1.317 ± 0.152	0.297	1.413 ± 0.205	1.338 ± 0.112	0.093	1.458 ± 0.221	1.399 ± 0.166	0.255	1.358 ± 0.239	1.397 ± 0.257	0.561
Post-VDT operation (s)	1.434 ± 0.203	1.437 ± 0.135	0.938	1.428 ± 0.214	1.443 ± 0.173	0.781	1.482 ± 0.231	1.447 ± 0.149	0.501	1.454 ± 0.276	1.442 ± 0.146	0.848
Change between pre- and post-VDT operation (s)	0.066 ± 0.115	0.120 ± 0.127	0.098	0.016 ± 0.156	0.104 ± 0.149	0.033	0.023 ± 0.177	0.048 ± 0.138	0.560	0.095 ± 0.141	0.046 ± 0.251	0.364

Data are presented as the mean ± SD. * Student’s *t*-test, vs the placebo group.

**Table 5:** The results of the MPOD levels.

	Baseline			2 Weeks			4 Weeks			8 Weeks		
	Active Group (*n* = 28)	Placebo Group (*n* = 29)	*p* Value *	Active Group (*n* = 28)	Placebo Group (*n* = 29)	*p* Value *	Active Group (*n* = 27)	Placebo Group (*n* = 29)	*p* Value *	Active Group (*n* = 28)	Placebo Group (*n* = 29)	*p* Value *
MPOD	0.576 ± 0.151	0.581 ± 0.136	0.904	0.587 ± 0.149	0.572 ± 0.151	0.712	0.567 ± 0.161	0.560 ± 0.156	0.855	0.591 ± 0.148	0.565 ± 0.145	0.502
Change of MPOD levels from 0 week				0.011 ± 0.052	−0.009 ± 0.065	0.220	−0.029 ± 0.136	−0.021 ± 0.066	0.781	0.015 ± 0.052	−0.016 ± 0.052	0.029

Data are presented as the mean ± SD. * Student’s *t*-test, vs the placebo group.

## Data Availability

Data is unavailable due to privacy or ethical restrictions.
